# Brain morphology of childhood aggressive behavior: A multi-informant study in school-age children

**DOI:** 10.3758/s13415-015-0344-9

**Published:** 2015-03-24

**Authors:** Sandra Thijssen, Ank P. Ringoot, Andrea Wildeboer, Marian J. Bakermans-Kranenburg, Hanan El Marroun, Albert Hofman, Vincent W. V. Jaddoe, Frank C. Verhulst, Henning Tiemeier, Marinus H. van IJzendoorn, Tonya White

**Affiliations:** 1 School of Pedagogical and Educational Sciences, Erasmus University Rotterdam, P.O. Box 1738, 3000 DR Rotterdam, The Netherlands; 2 The Generation R Study Group, Erasmus University Medical Center, Rotterdam, The Netherlands; 3 Department of Child and Adolescent Psychiatry/Psychology, Erasmus University Medical Center-Sophia Children’s Hospital, Rotterdam, The Netherlands; 4 Centre for Child and Family Studies, Leiden University, Leiden, The Netherlands; 5 Leiden Institute for Brain and Cognition (LIBC), Leiden University, Leiden, The Netherlands; 6 Department of Epidemiology, Erasmus University Medical Center, Rotterdam, The Netherlands; 7 Department of Pediatrics, Erasmus University Medical Center, Rotterdam, The Netherlands; 8 Department of Psychiatry, Erasmus University Medical Center, Rotterdam, Netherlands; 9 Department of Radiology, Erasmus University Medical Center, Rotterdam, The Netherlands

**Keywords:** Childhood aggression, Structural MRI, Sensorimotor cortex, Default mode network, Multiple-informant

## Abstract

**Objective:**

Few studies have focused on the neuroanatomy of aggressive behavior in children younger than 10 years. Here, we explored the neuroanatomical correlates of aggression in a population-based sample of 6- to 9-year-old children using a multiple-informant approach.

**Methods:**

Magnetic resonance (MR) scans were acquired from 566 children from the Generation R study who participated in the Berkeley Puppet Interview and whose parents had completed the Child Behavior Checklist. Linear regression analyses were used to examine associations between aggression and amygdala and hippocampal volume. We performed surface-based analyses to study the association between aggression and cortical thickness, surface area, and gyrification.

**Results:**

Aggressive behavior was associated with smaller amygdala (*p* < .05) but not hippocampal volume. Aggression was associated with a thinner cortex in the left precentral cortex (*p* < .01) and in a cluster including the right inferior parietal, supramarginal, and postcentral cortex (*p* < .001). Gender moderated the association between aggression and cortical thickness in the right medial posterior cortex (*p* = .001) and the right prefrontal cortex (*p* < .001). Aggression was associated with decreased gyrification in a large cluster including the right precentral, postcentral, frontal, and parietal cortex (*p* = .01). Moreover, aggression was associated with decreased gyrification in the right occipital and parietal cortex (*p* = .02).

**Conclusion:**

We found novel evidence that childhood aggressive behavior is related to decreased amygdala volume, decreased sensorimotor cortical thickness, and decreased global right hemisphere gyrification. Aggression is related to cortical thickness in regions associated with the default mode network, with negative associations in boys and positive associations in girls.

**Electronic supplementary material:**

The online version of this article (doi:10.3758/s13415-015-0344-9) contains supplementary material, which is available to authorized users.

## Introduction

There is accumulating evidence that aggressive behavior may be associated with specific neurobiological factors (Boes, Tranel, Anderson, & Nopoulos, 2008; Ducharme et al., 2011; Siever, 2008; Sterzer & Stadler, 2009). Imaging studies of adult and adolescent aggression have repeatedly reported associations with the amygdala, hippocampus, anterior cingulate cortex (ACC), and orbitofrontal cortex (OFC) (Siever, 2008). However, differences in brain functioning and structure related to adult aggression may be affected by the behavior itself (reversed causality) and/or environmental influences (e.g., lead exposure). Studying the neurobiological correlates of aggression in children increases the chance of identifying structures that are involved in the etiology of aggression (Sterzer & Stadler, 2009). Compared to adult and adolescent aggression, aggressive behavior in childhood has been relatively understudied. Imaging studies that have focused on childhood aggression have often examined the neurobiology of aggression in clinical samples. Examining the neuroanatomy of normal variation in aggressive behavior may provide information critical to our understanding of normal human behavior as well as psychopathology (Ducharme et al., 2011). Moreover, while there is general consensus that child behavior requires assessment from multiple informants (Kraemer et al., 2003), most neuroimaging studies on childhood aggression rely only on parent-reported aggression data (e.g. Ameis et al., 2014; Ducharme et al., 2011; Visser et al., 2014). In the present study, we explored the neuroanatomical correlates of normal variation in aggressive behavior in a large population-based sample of 6- to 9-year-old children using a multiple-informant approach.

Individuals expressing high levels of aggressive behavior may be viewed as having “a lower threshold for activation of motoric aggressive responses to external stimuli without adequate reflection or regard for the aversive consequences of the behavior” (p.340) (Siever, 2008). This may point to an imbalance between limbic drives, such as the amygdala, and prefrontal control mechanisms. Functional magnetic resonance imaging (fMRI) research has shown that aggressive adults display increased amygdala reactivity and decreased OFC activation in response to angry faces (Coccaro, McCloskey, Fitzgerald, & Phan, 2007). In highly aggressive individuals, diminished connectivity between the amygdala and the OFC has been observed (Coccaro et al., 2007). Moreover, the structure of the amygdala, OFC, ACC, and the dorsolateral prefrontal cortex (DLPFC) are also related to aggression (Schiffer et al., 2011; Yang & Raine, 2009).

In children, aggressive behavior has been studied in the context of two disorders characterized by high levels of aggression: conduct disorder (CD) and oppositional defiant disorder (ODD). Both disorders have been related to functional and structural abnormalities in the DLPFC, OFC, ACC, amygdala, and hippocampus (Fairchild et al., 2013; Fairchild et al., 2011; Gavita, Capris, Bolno, & David, 2012; Huebner et al., 2008; Matthys, Vanderschuren, & Schutter, 2013). Previous studies of children and adolescents have reported negative correlations between aggressive behavior and ACC and OFC thickness in boys and girls (Ameis et al., 2014; Boes et al., 2008; Ducharme et al., 2011; Walhovd, Tamnes, Ostby, Due-Tonnessen, & Fjell, 2012), and increased hippocampus volume in girls only (Visser et al., 2014). Gender differences in both aggression (Alink et al., 2006; Borsa, Damasio, Bandeira, & Gremigni, 2013) and neurobiological development (Mutlu et al., 2013; Raznahan et al., 2010) have been amply documented. There is also evidence that the neuroanatomic correlates underlying childhood aggression may show gender-related differences (Ducharme et al., 2011; Fairchild et al., 2013; Visser et al., 2014).

Previous normative neuroimaging studies on childhood aggression in non-clinical samples have mostly included samples covering a wide age range (±10 years) and/or a mean age above 10 years (Ameis et al., 2014; Ducharme et al., 2011; Visser et al., 2014; Walhovd et al., 2012). However, in childhood and young adolescence the brain is developing rapidly. In general, cortical gray matter development is characterized by a nonlinear growth curve, with gray matter volume reaching its peak around puberty (Giedd et al., 1999). Regions associated with more primary functions develop earlier compared to regions involved in more complex tasks (e.g., prefrontal and temporal cortex) (Gogtay et al., 2004). Since the cortical areas that are most often related to aggressive behavior reach their cortical peak after age 10, focusing on the brain of young children may provide information on the developmental neurobiology of childhood aggression that might otherwise go unnoticed. Moreover, many studies on the neuroanatomic correlates of aggression have focused on cortical gray matter volume. However, cortical volume is a function of cortical thickness and cortical surface area. Cortical thickness and surface area can be viewed as separate endophenotypes, reflecting the underlying genetic influence on brain development (Rakic, 1995). Cortical gyrification (cortical folding) is an important property of the cortex that helps to increase cortical surface area within a confined space (Reillo et al., 2011). Focusing on cortical thickness and cortical area separately and *a*dditionally examining cortical gyrification provides complementary information on the brain morphology of childhood aggression.

The present study assessed the association between the normal variation in aggressive behavior and cortical thickness, surface area, gyrification, and amygdala and hippocampal volume in a large population-based sample of 6- to 9-year-old children using combined parent-reported and child-reported measures of aggression. We hypothesized that aggression would be related to reduced amygdala and hippocampal volume. Although previous studies strongly imply an association between the prefrontal cortex and aggression, this association may not yet be fully present in 6- to 9-year-olds. Based on prior studies showing gender differences in aggressive behavior as well as cortical development, we also expected gender-related differences in the association between brain morphology and aggression.

## Methods

### Participants

Participants were recruited from the Generation R study, a population-based prospective cohort from early fetal life onwards in Rotterdam, the Netherlands (Jaddoe et al., 2012; Tiemeier et al., 2012). From 799 eligible 6- to 9-year-old children with structural imaging data, 117 (15 %) children were missing parent- and/or child-reported aggression scores. From the remaining 682 children, 90 (11 %) had poor quality T1 data. Seven twin pairs were excluded from the analyses and from seven sibling pairs, one child was randomly excluded. As our aggression measures were suitable for children younger than 8 years, five children were excluded who fell above this cut-off. Consequently, cortical surface analyses were performed on 566 children (285 boys).

This study was approved by the Medical Ethics Committee of the Erasmus Medical Centre, Rotterdam. All parents provided informed consent.

### Measures

*Aggressive behavior* – *parent report* The primary caregiver completed the Child Behavior Checklist 1 ½ - 5 years (CBCL 1 ½ - 5) (Achenbach & Rescorla, 2000) when the children were on average 6 years old. We chose to use the CBCL 1 ½ - 5 to enable the use of the same measure of parent-reported aggressive behavior for the entire sample. Although the average age of the sample was 6 years at time of the aggression assessment, the majority of the children (60 %) were still 5 years of age. CBCL can be scored on eight scales, including the Aggressive Behavior scale that was used in the present study (*M* = 5.38, *SD* = 4.68). This scale consists of 19 items scored on a three-point Likert scale (α = .87). To approach normality, Aggressive Behavior scores were square-root transformed (Tabachnick & Fidell, 2007).

*Aggressive behavior* – *child report* Self-reported aggressive behavior measures were obtained using the Berkeley Puppet Interview (BPI) (Ablow, Measelle, & Assessment, 2003) when the children were on average 6 years old. Details on BPI data collection in Generation R have been described elsewhere (Ringoot et al., 2013). Briefly, two identical hand puppets named “Iggy” and “Ziggy” presented the children with opposing statements. The child was asked to indicate which statement described him or her best. Videotaped responses were scored on a seven-point Likert scale (intraclass correlation for intercoder reliability .96–.98), with higher scores representing more problems.

We used the broadband Externalizing scale – including the Oppositional Defiant, Overt Hostility and Conduct Problems scales – as an indicator of aggressive behavior. We chose to use this scale (α = .77, *M* = 51.69, *SD* = 10.33) as it was most comparable to the CBCL Aggressive Behavior scale. For example, CBCL Aggressive Behavior items such as “Screams” or “Disobedient” reflect Oppositional Defiant items in the BPI (“When I don’t get my own way, I don’t yell at my teacher/mom or dad” “I do what my teacher asks me to do”). Items such as “Hits others,” “Attacks people,” or “Destroys others’ things” reflect items in the BPI Overt Hostility and Conduct Problems scales (“I don’t hit my mom or dad/my teacher, or other grown-ups,” “I don’t fight with other kids,” “I don’t break other peoples’ things when I’m mad at them”). To approach normality, the Externalizing scale was inverse transformed (Tabachnick & Fidell, 2007) and then standardized to increase interpretability.

*Combining parent and child reported data* In order to obtain a multiple-informant aggressive behavior score, we aggregated scores reported by the parent and the child. Parent and child scores showed a correlation of *r* = .12. Generally, agreement between parent and child informants tends not to exceed 0.20 (Kraemer et al., 2003). Low levels of agreement between informants suggest that childhood functioning is best conceptualized as the separate and combined influences of children’s actual characteristics, the context in which children are observed, the perspectives (or biases) of the informants, and error of measurement. Kraemer et al. (2003) therefore suggest that the choice of informants should be based on consideration of the contexts and perspectives that influence the characteristic under investigation. Weaknesses of one informant should be compensated by strengths of another. While parents observe their child only in the home environment, the child itself can report on his or her behavior in all contexts. Furthermore, children provide information on how they perceive themselves, while parents provide an other-report. As recommended by Kraemer et al. (2003), we performed an unrotated principal component analysis on the items of the two scales. The first component provides a multiple-informant measure of aggression that is relatively free of informant bias, whereas the second component is thought to reflect rater differences. We also created a multiple-informant aggression score by calculating the mean of the standardized untransformed parent and child scores. Since the correlation between the averaged multiple-informant aggression score and the multiple-informant component coefficient was extremely high (*r* = .93), we decided to use the averaged aggression score for further analyses. The associations between the multiple-informant score and the parent and child reported scores each amounted to *r* = .75. The multiple-informant aggression score was square-root transformed to approach normality.

*Covariates* Information on gender, gestational age, and date of birth was obtained from midwives and hospital registries. Ethnicity, maternal education level and smoking during pregnancy were assessed through questionnaires. Handedness was measured using a modified version of the Edinburgh Handedness Inventory (Oldfield, 1971). Intelligence (IQ) was estimated from the Mosaics and Categories subtest of the Snijders-Oomen Non-Verbal Intelligence Test-Revised (Tellegen, Winkel, Wijnberg-Williams, & Laros, 2005). The prosocial scale of the Strengths and Difficulties Questionnaire (SDQ) was used as a measure of prosocial behavior (Goodman, 1997; Paap et al., 2013). Attention problems and internalizing problems were measured using the CBCL (Achenbach & Rescorla, 2000). Parental psychopathology was measured using the anxiety and depression scales of the Brief Symptom Inventory (Derogatis & Melisaratos, 1983). Models were adjusted for covariates that generated a change in predictor effect estimates of 5 % or more.

*Structural magnetic resonance imaging* A description of the neuroimaging component of the Generation R study has been described elsewhere (White et al., 2013). Children were 6–9 years of age at the time of the MRI assessment. On average, time between aggressive behavior assessment and the MRI was 1.76 years. Prior to the MRI, the children were first familiarized with a mock scanning session. MRI scanning was performed on a GE Discovery MR 750 3 T scanner (General Electric, Milwaukee, MI, USA). T_1_-weighted inversion recovery fast spoiled gradient recalled (IR-FSPGR) sequence was obtained using an 8-channel head coil with the following parameters: TR = 10.3 ms, TE = 4.2 ms, TI = 350 ms, NEX = 1, flip angle = 16°, readout bandwidth = 20.8 kHz, matrix 256x256, imaging acceleration factor 2, and an isotropic resolution of 0.9x0.9x0.9 mm^3^.

### Image processing

Cortical reconstruction and volumetric segmentation was performed with the FreeSurfer image analysis suite 5.1 (http://surfer.nmr.mgh.harvard.edu/). The technical details of these procedures are described in prior publications (Reuter, Schmansky, Rosas, & Fischl, 2012). Briefly, this process included the removal of non-brain tissue, automated Talairach transformation into standard space, and segmentation of the subcortical white and gray matter volumetric structures (including the amygdala and hippocampus), intensity normalization, tessellation of the gray matter white matter boundary, automated topology correction, and surface deformation.

Once the cortical models were complete, the images underwent surface inflation (Fischl, Sereno, & Dale, 1999), registration to a spherical atlas (Fischl, Sereno, Tootell, & Dale, 1999), and the parcellation of the cerebral cortex into units based on gyral and sulcal structure (Desikan et al., 2006). Cortical thickness was calculated as the closest distance from the gray/white boundary to the gray/cerebrospinal fluid boundary at each vertex on the tessellated surface (Fischl & Dale, 2000). The surface-based map was smoothed with a 10-mm full-width half-maximum (FWHM) Gaussian kernel prior to the surface-based analyses. Several studies using Freesurfer in typically and atypically developing school-aged children are available (El Marroun et al., 2014; Juuhl-Langseth et al., 2012).

To assess the local gyrification index (LGI) we used the method of Schaer et al. (2008), which is implemented in FreeSurfer. This approach provides an estimation of the local gyrification index, taking into account the three-dimensional cortical surface. Identification of the pial and white matter surfaces against an additional surface that tightly wraps the pial surface are used to estimate the degree of cortical folding at a 25-mm spherical vertex-based region. This method has been validated and used in several studies focusing on childhood and adolescent psychopathology (Schaer et al., 2013; Wallace et al., 2014). The surface-based LGI maps were smoothed prior to the analyses using a 5-mm FWHM Gaussian kernel, consistent with comparable studies (Wallace et al., 2014). Gyrification data was available on 557 children.

### Image quality

All unprocessed T1 images were visually inspected at the scan site. Raters were instructed to assess movement and scanner artifacts on a six-point scale (unusable, poor, fair, good, very good, excellent). After processing through the FreeSurfer pipeline, overall segmentation quality of the images was inspected on a seven-point scale (not constructed, poor, fair, fairly good, good, very good, excellent). Unprocessed images rated as unusable or poor were excluded from the analyses (n = 31), as were images that could not be processed by FreeSurfer and images with a poor segmentation quality (n = 59). We additionally rated amygdala and hippocampal segmentation quality as usable or unusable. Scans with unusable hippocampal or amygdala segmentation quality (n = 66) were excluded from the amygdala and hippocampal analyses only.

### Statistical analyses

Missing values on the covariates (1–23 %) were imputed using the predictive mean matching method. Results of ten imputed datasets were pooled to obtain an overall outcome.

Hierarchical linear regression analyses were used to examine the association between aggressive behavior and amygdala and hippocampal volume. Amygdala and hippocampal volume were residualized for total brain volume (TBV). In the first block, gender, age, IQ, and aggression were entered. In the second step, the covariates were entered (gestational age, ethnicity, image quality, attentional problems, internalizing problems, prosocial behavior, maternal education, and maternal smoking during pregnancy), and in the third step the gender-by-aggression interaction term was entered.

We performed vertex-wise exploratory analyses of the association between aggression and cortical thickness, cortical surface area, and cortical gyrification across the entire cortex. Furthermore, we examined whether gender moderated the association between cortical thickness, surface area, or gyrification and aggression. For this purpose, FreeSurfer’s QDEC was used (www.surfer.nmr.mgh.harvard.edu). Regions for which cortical thickness, surface area, or gyrification were significantly associated with aggressive behavior as well as regions in which gender moderated this association were determined using general linear models (GLMs) with age, gender, and IQ as covariates. For use in QDEC, missing values on intelligence were mean imputed. To correct for the effect of multiple comparisons, a cluster-based Monte Carlo simulation was performed using 10,000 iterations and *p* ≤ .05.

Surface-based Regions Of Interest (ROIs) were manually created for brain areas that were significantly related to aggression and utilized to extract the mean cortical thickness, surface area, or gyrification within the specific ROI for every participant. ROIs were then residualized for TBV. Hierarchical linear regression analyses were used to examine the effect of confounding variables on the extracted ROI (gender, age, IQ, and aggression in the first block, covariates in the second). Age-by-ROI and gender-by-age-by-ROI interaction effects were also tested. As these effects were not significant, they are not reported.

To confirm our primary findings, we bootstrapped the GLM analysis using 500 iterations of 200 participants (Efron & Tibshirani, 1993). For each voxel, *p*-values were averaged to generate an overall result. Moreover, analyses were repeated for CBCL Aggressive Behavior and BPI Externalizing Behavior scores separately. These results are provided as Supplemental Material (Supplementary Text 1, Supplementary Table 1 and Supplementary Figs. 1 and 2).

## Results

Demographic characteristics of the sample can be found in Table 1. For all assessments, boys and girls were comparable in age (*p*s > .15). Boys had higher aggression scores than girls (*F*(1,564) = 11.64, *p* = .001, *d* = 0.28 for CBCL, *F*(1,564) = 47.62, *p* < .001, *d* = 0.57 for BPI, and *F*(1,570) = 45.93, *p* < .001, *d* = 0.57 for the multiple-informant aggression score). Age at assessment was not related to aggressive behavior. There were negative correlations between IQ and aggressive behavior using the multiple-informant aggression score, *r* = −.12, *p* < .01, the parent-reported CBCL *r* = −.10, *p* = .03, and the child-reported BPI *r* = −.08, *p* = .06. Prosocial behavior was negatively related to aggressive behavior for the multiple-informant score, *r* = −.21, *p* < .001, and for the parent-reported CBCL *r* = −.24, *p* < .001, but not for the child-reported BPI *r* = −.05, *p* = .30.

**Table 1:** Demographic characteristics

N = 566	*M* (*SD*) or n (%)
Boys	285 (50.4 %)
Ethnicity	
Dutch	433 (76.5 %)
Other-Western	33 (5.8 %)
Non-Western	100 (17.7 %)
Gestational age	40.06 (1.63)
IQ	104.08 (14.02)
Right-handed	517 (90.5 %)
SDQ Prosocial Behavior	13.33 (1.79)
CBCL Aggressive Behavior	5.38 (4.68)
Age CBCL	6.03 (0.41)
CBCL filled out by	
Mother	521 (91.8 %)
Father	37 (6.5 %)
Both	6 (1.1 %)
Other	1 (0.2 %)
BPI Externalizing	51.69 (10.33)
Age BPI	6.11 (0.39)
Age MRI	7.87 (0.97)
Maternal Education	
Primary or lower	11 (1.9 %)
Secondary	213 (37.6 %)
Higher	333 (58.8 %)
Maternal smoking during pregnancy	
Never	437 (77.2 %)
Quit when pregnancy known	34 (6.0 %)
Continued	91 (16.1 %)
Parental psychopathology	
Depression	1.34 (2.53)
Anxiety	1.47 (2.31)

### Association between aggressive behavior and amygdala and hippocampal volume

Table 2 shows the associations between aggressive behavior and amygdala and hippocampal volume. Aggression was associated with a smaller amygdala volume, β = −.12, *p* < .05 for total amygdala volume, and β = −.11, *p* = .03 and β = −.08, *p* = .11, for right and left amygdala volume, respectively. Aggressive behavior was not associated with hippocampal volume, β = −.02, *p* = .76. Scatterplots of the association between aggressive behavior and amygdala and hippocampal volume are provided in Supplementary Fig. 3.

**Table 2:** Associations between aggressive behavior and amygdala and hippocampal volume (n = 500)

		Total			Left			Right		
		B (95 % CI)	β	*part r*	B (95 % CI)	β	*part r*	B (95 % CI)	β	*part r*
Amygdala	Baseline adjusted^1^	−93.14 (−201.12; 14.84)	−.08	−.06	−34.62 (−91.25; 22.01)	−.06	−.05	−54.38 (−119.99; 11.24)	−.08	−.07
	Adjusted^2^	−136.11 (−259.03; −13.18)	−.12*	−.10	−52.16 (−116.39; 12.07)	−.08	−.07	−81.09 (−155.95;−6.23)	−.11*	−.10
Hippocampus	Baseline adjusted^1^	39.76 (−124.68; 204.20)	.02	.02	49.42 (−40.68; 139.51)	.05	.05	−11.73 (−103.03; 79.57)	−.02	−.01
	Adjusted^2^	−29.47 (−216.84; 157.90)	−.02	−.01	5.50 (−96.63; 107.64)	.01	.01	−35.43 (−139.83; 68.96)	−.03	−.03

*Note*. * p < .05. Amygdala and hippocampus volume were corrected by TBV

^1^Adjusted for gender and age

^2^Adjusted for gender, age, IQ, gestational age, ethnicity, handedness, attention problems, internalizing problems, prosocial behavior, image quality, maternal education, parental psychopathology, and maternal smoking during pregnancy

### Association between aggressive behavior and cortical thickness

**Fig. 1:**
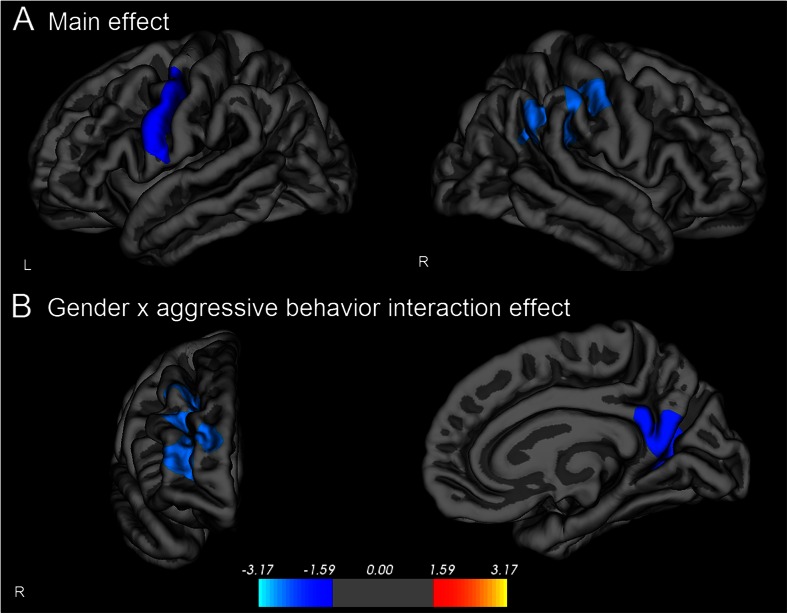
Relation between cortical thickness and aggressive behavior. Sex, age, and IQ were used as covariates (Monte Carlo corrected cluster-wise *p* < .05). **a**) Cortical thickness was negatively associated with aggression in a cluster including the left precentral cortex and a cluster including the right inferior parietal, supramarginal, and postcentral cortex (i.e., reduced cortical thickness was associated with more aggressive behavior). **b**) A moderating effect of gender was found for a cluster including the right middle frontal, and superior frontal cortex and for a cluster including the right precuneus, isthmus of the cingulate cortex, and lingual cortex, with negative associations in boys and positive associations in girls. Colors represent –log10 *p*-value

Aggressive behavior was associated with reduced cortical thickness in a cluster including the left precentral cortex (1,150 mm^2^, max vertex X = −52.5, Y = −6.6, Z = 38.5, *p* = .005) and a cluster including the right inferior parietal, supramarginal, and postcentral cortex (2,139 mm^2^, max vertex X = 42.9, Y = −27.6, Z = 37.6, *p* < .001, Fig. 1a). Gender moderated the association between aggression and cortical thickness in a cluster including the right precuneus, isthmus of the cingulate cortex, and lingual cortex (1,344 mm2, max vertex X = 8.5, Y = −53.1, Z = 20.4, *p* = .001), as well as in a cluster covering the right middle and superior frontal cortex (2,067 mm^2^, max vertex X = 34.4, Y = 49.8, Z = 7.7, *p* < .001, Fig. 1b).

To examine the effect of possible confounding variables on the association between the ROIs and aggression, hierarchical linear regression models were used. Greater aggressive behavior was associated with reduced cortical thickness of the left precentral ROI and the right postcentral ROI, β = -.13, *p* = .005 and β = −.15, *p* = .001 respectively (Table 3). For regions in which there was a significant gender-by-aggression interaction effect, analyses were repeated for boys and girls separately (Table 4). In girls, aggressive behavior was associated with a thicker cortex in the right precuneus ROI and the right frontal ROI , β = .16, *p* = .02 and β = .22, *p* < .001 respectively. In boys, aggression was not related to the right precuneus ROI, β = -.12, *p* = .07. In the right frontal ROI, we found a negative association between cortical thickness and aggressive behavior in boys, β = −.14, p = .04. To assure that the gender-by-aggression interaction effect was not caused by gender differences in mean levels of aggression, we repeated the analysis on a subset of 190 boys and 190 girls with equivalent aggression scores. Results were similar. To confirm our primary findings, we bootstrapped the GLM analysis using 500 iterations of 200 participants. Supplementary Fig. 4 shows a cortical map of the averaged *p*-values across the 500 bootstraps. Although cluster size may differ, all clusters were confirmed.

**Table 3:** Associations between aggressive behavior and cortical thickness (n = 566)

		B (95 % CI)	β	*part r*
L precentral ROI	Baseline adjusted^1^	−0.10 (−0.15;−0.04)	−.15***	−.14
	Adjusted^2^	−0.09 (−0.15;−0.03)	−.13**	−.11
R postcentral ROI	Baseline adjusted^1^	−0.11 (−0.16;−0.07)	−.20***	−.19
	Adjusted^2^	−0.09 (−0.14;−0.04)	−.15**	−.13

*Note*. ** p < .01*** p < .001. Cortical thickness was corrected by TBV

^1^Adjusted for age, IQ, and gender

^2^Adjusted for age, gender, IQ, ethnicity, attention problems, internalizing problems, prosocial behavior, image quality, and maternal smoking during pregnancy

**Table 4:** Gender moderating effects in cortical thickness ROIs (n = 566)

		Aggression x gender			Boys			Girls		
		B (95 % CI)	β	*part r*	B (95 % CI)	β	*part r*	B (95 % CI)	β	*part r*
R precuneus ROI	Baseline adjusted^1^	−0.29 (−0.44;−0.14)	−.24***	−.15	−0.10 (−0.20;−0.01)	−.13*	−.12	0.17 (0.05;0.29)	.17**	.17
	Adjusted^2^	−0.27 (−0.42;−0.12)	−.23***	−.15	−0.10 (−0.21;0.01)	−.12	−.10	0.17 (0.02;0.31)	.16*	.13
R frontal ROI	Baseline adjusted^1^	−0.21 (0.27;−0.16)	−.25***	−.16	−0.09 (−0.16;−0.01)	−.14*	−.14	0.13 (0.05;0.21)	.19**	.19
	Adjusted^2^	−0.21 (−0.31;−0.11)	−.25***	−.16	−0.09 (−0.16;0.01)	−.14*	−.12	0.15 (0.07;0.24)	.22***	.19

*Note*. * p < .05 ** p < .01*** p < .001. Cortical thickness was corrected by TBV

^1^Adjusted for age, IQ (and gender)

^2^Adjusted for age, IQ, (gender), ethnicity, attention problems, internalizing problems, prosocial behavior, image quality, and maternal smoking during pregnancy

### Association between aggressive behavior and cortical surface area

The surface-based whole brain analysis of the association between cortical surface area and aggressive behavior provided no significant results.

### Association between aggressive behavior and gyrification

**Fig. 2:**
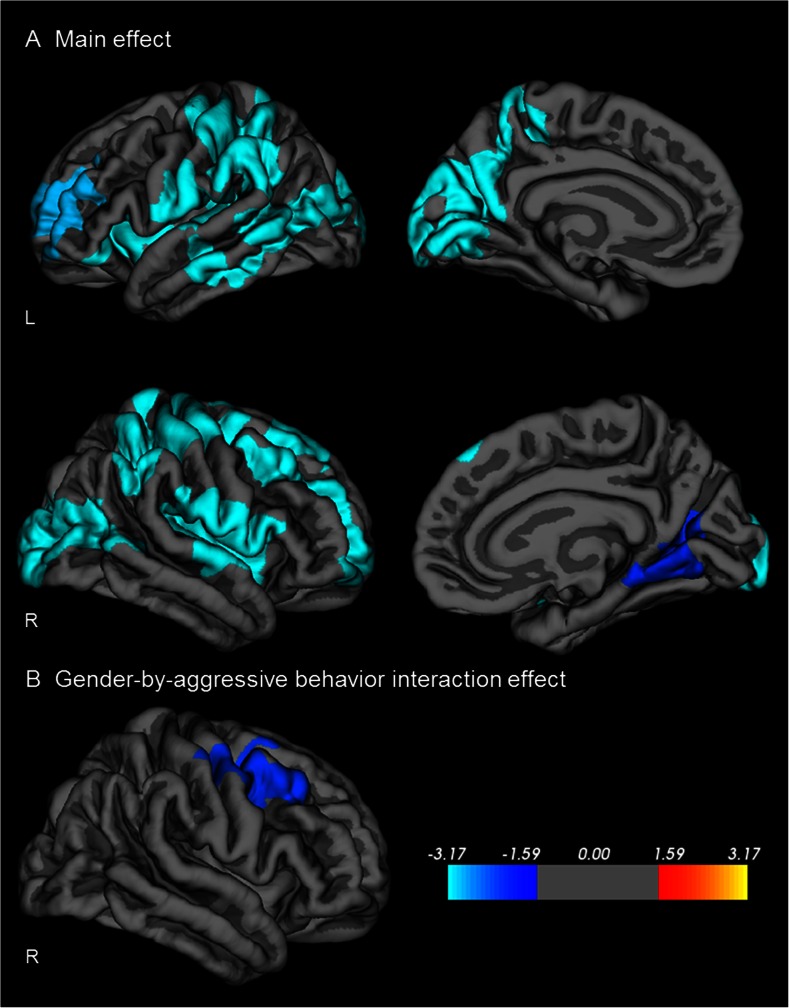
Relation between cortical gyrification and aggressive behavior. Sex, age, and IQ were used as covariates (Monte Carlo corrected cluster-wise *p* < .05). **a**) Cortical gyrification was negatively associated with aggression in a cluster including the left precentral cortex, extending to the postcentral, paracentral, parietal, temporal, occipital, precuneus, and inferior frontal cortex as well as in a cluster including the left rostral middle frontal cortex (i.e.reduced gyrification was associated with more aggressive behavior). In the right hemisphere, aggressive behavior was associated with reduced gyrification in a cluster including the precentral cortex, extending anteriorly to the frontal cortex, and posteriorly to postcentral, and parietal cortex. The second right hemisphere cluster included the postcentral and insular cortex. The third right hemisphere cluster included the lateral occipital and inferior parietal cortex, while the forth cluster included the lingual cortex, extending to the precuneus and cuneus. **b**) A moderating effect of gender was found for a cluster including the right middle frontal, and superior frontal cortex and for a cluster including the right right precentral, postcentral, frontal, and supramarignal cortex. In this region, greater aggressive behavior was associated with reduced gyrification in boys only. Colors represent –log10 *p*-value

Figure 2 shows the association between aggressive behavior and gyrification. Aggressive behavior was associated with decreased gyrification in a cluster including the left precentral cortex, extending to the postcentral, paracentral, parietal, temporal, occipital, precuneus, and inferior frontal cortex (24,314 mm^2^, max vertex X = −36.8, Y = −18.3, Z = 64.5, *p* < .001), as well as in a cluster including the left rostral middle frontal cortex (3,010 mm^2^, max vertex X = −22.1, Y = 41.5, Z = 24.1, *p* = .001). In the right hemisphere, aggressive behavior was associated with deceased gyrification in a cluster including the precentral cortex, extending anteriorly to the middle and superior frontal cortex, and posteriorly to postcentral, superior parietal, and supramarginal cortex (11,807 mm^2^, max vertex X = 27.7, Y = −14.4, Z = 60.2, *p* = .001). Moreover, we found a negative association between aggression and gyrification in a cluster including the postcentral, and insular cortex (6,060 mm^2^, max vertex X = 62.1, Y = −9.6, Z = 28.9, *p* = .001), as well as in a cluster including the lateral occipital and inferior parietal cortex (5,355 mm^2^, max vertex X = 21.3, Y = −98.7, Z = 5.3, *p* = .001), and in a cluster including the lingual cortex, extending to the precuneus and cuneus (2,568 mm^2^, max vertex X = 25.4, Y = −61.6, Z = 0.7, *p* = .01). Gender moderated the association between aggressive behavior and gyrification in a cluster including the right precentral, postcentral, and caudal middle frontal cortex (5,762 mm^2^, max vertex X = 27.7, Y = −14.4, Z = 60.2, *p* < .001). This cluster overlaps with the right hemisphere main effect precentral cluster.

Results of the hierarchical regression analyses correcting for total brain volume and confounding variables can be found in Table 5. After correction for confounding variables, aggression was associated with decreased gyrification only in the right precentral ROI and right lateral occipital ROI, β = −.13, *p* = .01 and β = −.10, *p* = .02, respectively. As the right precentral ROI overlaps with the gender-by-aggression interaction ROI, we additionally tested a gender-by-aggression interaction term. This interaction term was not significant, β = −.11, *p* = .10. For the right gender-by-aggression interaction ROI analyses were repeated for boys and girls separately (Table 6). In this region, more aggressive behavior was associated with reduced gyrification in boys only, β = −.19, *p* = .006. To assure that the gender-by-aggression interaction effect was not caused by gender differences in mean levels of aggression, we repeated the analysis on a subset of 190 boys and 190 girls with equivalent aggression scores. Results were similar.

**Table 5:** Associations between aggressive behavior and gyrification (n = 557)

		B (95 % CI)	β	*part r*
L precentral ROI	Baseline adjusted^1^	−0.04 (−0.07; −0.00)	−.09*	−.09
	Adjusted^2^	−0.03 (−0.07; 0.01)	−.06	−.05
L rostral middle frontal ROI	Baseline adjusted^1^	−0.04 (−0.07; 0.00)	−.09	−.08
	Adjusted^2^	−0.03 (−0.07; 0.01)	−.08	−.07
R precentral ROI	Baseline adjusted^1^	−0.04 (−0.08; −0.00)	−.09*	−.09
	Adjusted^2^	−0.06 (−0.10; −0.01)	−.13*	−.11
R postcentral ROI	Baseline adjusted^1^	−0.03 (−0.11; 0.05)	−.03	−.03
	Adjusted^2^	0.02 (−0.07; 0.11)	.02	.02
R lateral occipital ROI	Baseline adjusted^1^	−0.05 (−0.09; −0.01)	−.12**	−.11
	Adjusted^2^	−0.04 (−0.09; −0.00)	−.10*	−.08
R lingual ROI	Baseline adjusted^1^	−0.06 (−0.13; 0.01)	−.07	−.07
	Adjusted^2^	−0.04 (−0.11; 0.04)	−.05	−.04

*Note*. * p < .05 ** p < .01. Gyrification was corrected by TBV.

^1^Adjusted for age, IQ and gender

^2^Adjusted for age, IQ, gender, ethnicity, attention problems, internalizing problems, prosocial behavior, image quality, maternal smoking during pregnancy

**Table 6:** Gender moderating effect on gyrification of right precentral ROI (n = 557)

	Aggression x gender			Boys			Girls		
	B (95 % CI)	β	*part r*	B (95 % CI)	β	*part r*	B (95 % CI)	β	*part r*
Baseline adjusted^1^	−0.14 (−0.19; −0.08)	−.17*	−.11	−0.08 (−0.16; −0.01)	−.13*	−.13	0.04 (−0.03; 0.11)	0.07	.07
Adjusted^2^	−0.13 (−0.24; −0.03)	−.17*	−.11	−0.12 (−0.20; −0.03)	−.19**	−.17	0.04 (−0.04; 0.12)	0.07	.06

*Note*. * p < .05 ** p < .01. Gyrification was corrected by TBV

^1^Adjusted for age, IQ (and gender)

^2^Adjusted for age, IQ, (gender), ethnicity, attention problems, internalizing problems, prosocial behavior, image quality, and maternal smoking during pregnancy

To confirm our primary findings, we bootstrapped the GLM analysis using 500 iterations of 200 participants. Supplementary Fig. 5 shows a cortical map of the averaged *p*-values across the 500 bootstraps. All clusters were confirmed.

## Discussion

The present study examined the neuroanatomical correlates of aggressive behavior in 6- to 9-year-old children using a multiple-informant approach. As hypothesized, childhood aggression was associated with smaller amygdala volume. Moreover, aggressive behavior was associated with decreased cortical thickness in the left precentral cortex and the right inferior parietal, supramarginal and postcentral cortex. We found a moderating effect of gender on the association between aggressive behavior and cortical thickness in the right frontal cortex as well as in the right medial posterior cortex. While aggressive behavior was not associated with cortical surface area, we found widespread associations between aggressive behavior and decreased right hemisphere gyrification. Results were comparable across reporters.

Several studies have reported decreased amygdala volume in relation to conduct disorder, antisocial personality disorder, and psychopathy (Fairchild et al., 2011; Huebner et al., 2008; Pardini, Raine, Erickson, & Loeber, 2014). In the present study, aggressive behavior was associated with a smaller amygdala volume in typically developing children. Thus, amygdala volume may be associated with aggressive behavior along a continuum within the general population. Only two studies have examined the relation between normal variation in child or adolescent aggression and amygdala volume, and they did not find an association (Ameis et al., 2014; Visser et al., 2014). In our large sample, the association between aggression and amygdala volume was significant, but the effect size is small. Amygdala volume differences related to aggression may be more pronounced in clinical populations, but our findings show that the same association can be found in non-clinical groups.

Increased aggression was associated with decreased precentral, as well as inferior parietal, supramarginal, and postcentral cortical thickness. Although the precentral cortex – which is involved in motor planning and execution – has not typically been associated with aggressive behavior, several recent studies have found a relation with aggression. Precentral cortical thinning has been reported in association with psychopathy and violence (Ly et al., 2012; Narayan et al., 2007). Furthermore, activation of the precentral cortex has been associated with impulsivity in juvenile offenders and impaired response inhibition in highly aggressive male students (Pawliczek et al., 2013; Shannon et al., 2011). Structural differences of the postcentral cortex, also known as the somatosensory cortex, and the inferior partial lobule (which comprises the supramarginal and inferior parietal cortex) have previously been reported in relation to adolescent conduct disorder as well as adult violence and antisocial behavior (Aoki, Inokuchi, Nakao, & Yamasue, 2014; Hyatt, Haney-Caron, & Stevens, 2012; Narayan et al., 2007; Tiihonen et al., 2008). Interestingly, mirror neurons have been found in the precentral cortex, postcentral cortex, and inferior parietal lobule (Dushanova & Donoghue, 2010; Rizzolatti & Craighero, 2004). Mirror neurons are involved in understanding actions performed by others (Rizzolatti & Craighero, 2004). Cortical thinning in these sensorimotor regions may lead to a deficit in the understanding of others, which may cause the child to respond with aggression.

As expected, boys were more aggressive than girls (Alink et al., 2006; Borsa et al., 2013). We found a moderating effect of gender on the association between aggressive behavior and cortical thickness in a cluster covering the right precuneus, isthmus of the cingulate cortex, and lingual cortex as well as in a cluster covering the right middle and superior frontal cortex. For the right medial posterior cluster, cortical thickness was positively correlated with aggressive behavior in girls, but unrelated in boys. For the right frontal cluster, a thicker cortex was related to more aggression in girls, whereas in boys, thinning of the cortex was related to more aggressive behavior. While we did not expect to find opposite results between boys and girls, a study on brain morphology of conduct disorder in adolescence has also reported opposite findings between the sexes (Fairchild et al., 2013). These opposite correlations suggest that smaller scale studies on the neurobiology of aggression should be cautious when combining males and females.

Recently, thinning of the precuneus and posterior cingulate cortex (which includes the isthmus of the cingulate cortex) has been reported in adolescents diagnosed with ODD or CD (Fahim et al., 2011; Hyatt et al., 2012; Wallace et al., 2014). Thickness of the rostral middle frontal and superior frontal cortex has been related to non-clinical conduct problems in children (Walhovd et al., 2012), and a recent meta-analysis of adult antisocial and violent behavior found that the structure and function of the DLPFC, which occupies the middle frontal gyrus, is related to aggressive behavior (Yang & Raine, 2009). In adolescent girls with conduct disorder, the volume of the DLPFC was negatively correlated with aggressive conduct disorder symptoms (Fairchild et al., 2013). The precuneus, the posterior cingulate cortex, and the rostral middle frontal and superior frontal cortex are part of the default mode network (DMN) (Whitfield-Gabrieli & Ford, 2012). Activity of the DMN has been related to self-reflection, social perspective taking, moral decision-making, and future thought (Andrews-Hanna, 2012). Differences in cortical thickness in areas of the DMN may therefore be related to aggression either directly or indirectly through difficulties in self-reflection, perspective taking, and moral decision-making. In a study of default mode network connectivity in conduct disordered adolescent males with comorbid substance use disorder, Dalwani et al. (2014) found reduced activity in the middle frontal cortex, superior frontal cortex, and lingual cortex in patients compared to controls. DMN activity in the precuneus, posterior cingulate cortex, and lingual cortex was related to risk-taking behavior (Dalwani et al., 2014). Investigating the role of the DMN function in relation to childhood aggression is an important area for future studies.

Childhood aggression was associated with decreased gyrification in a cluster including the right precentral cortex, extending posteriorly to the postcentral, and parietal cortex, and anteriorly to the middle and superior frontal cortex. In part of this cluster, the negative association between gyrification and aggression was found only in boys. Moreover, aggressive behavior was related to decreased gyrification in a cluster including the right lateral occipital and inferior parietal cortex. These regions partly overlap with our cortical thickness findings, and thus suggest a general rather than specific structural relation with aggressive behavior. While the association between aggressive behavior and cortical thickness was restricted to relatively small clusters, aggression-related differences in gyrification were found across the entire lateral right hemisphere and thus suggest a more global effect. Considering this global effect, we tested whether the findings were also a reflection of global psychopathology. However, the associations between aggressive behavior and right hemisphere gyrification remained significant after correction for internalizing and attention problems. This provides evidence that our findings are specific markers of childhood aggression. As cortical gyrification shows its greatest growth during the third trimester of pregnancy, differences in gyrification may represent the consequences of early adverse events (White, Su, Schmidt, Kao, & Sapiro, 2010).

Our cortical thickness and gyrification findings suggest that (part of) the neuroanatomical correlates of aggression may be gender-specific. Sex hormones such as testosterone have been found to affect both brain development and aggressive behavior and may thus provide an explanation for these gender-specific findings (Cunningham, Lumia, & McGinnis, 2013). Alternatively, the effect found in cortical thickness may represent a difference in maturation (Thijssen, Wildeboer, Muetzel, Bakermans-Kranenburg, El Marroun, & Hofman et al., 2015). Previous studies show that the social brain matures faster in girls than in boys (Mutlu et al., 2013). Moreover, several studies have suggested that children with elevated psychiatric traits (e.g., conduct problems) have a delayed or aberrant cortical maturation (De Brito et al., 2009; Dennis & Thompson, 2013). In childhood, cortical thickness increases until it reaches its peak thickness around puberty (Giedd et al., 1999). Thereafter, processes such as synaptic pruning result in cortical thinning. If the girls in our sample already show cortical thinning, the positive association between cortical thickness and aggression may be explained by delayed maturation in high aggressive girls. Indeed, in boys 3 years older than the children examined here, De Brito et al. (2009) found greater OFC, ACC, and temporal lobe volume in boys with conduct problems and callous-unemotional traits compared to typically developing boys. Their post-hoc analyses indicate that their typically developing sample shows cortical decrease over age, while the boys with conduct problems either show an increase of volume over age or no relation between cortical volume and age. Due to their slower maturation compared to girls the boys in our sample may still show cortical increase. If the boys with high levels of aggressive behavior show a delayed cortical maturation, they will have a thinner cortex compared to the low aggressive boys. However, the present study presents cross-sectional data and thus does not provide direct information on neurodevelopmental trajectories underlying aggressive behavior. Longitudinal studies are necessary to shed light on the neurodevelopmental trajectories of aggression.

Although the hippocampus has often been implicated in aggressive behavior, we did not find an association between aggression and hippocampal volume. In their study on the neuroanatomy of normal variation in aggressive behavior, Visser et al. (2014) report an association between typically developing female adolescents’ aggression and hippocampus volume, but in the opposite direction compared to previous research: *increased* hippocampal volume was related to aggression in girls. As reduced hippocampal volume has been related to aggressive behavior in individuals suffering from psychological disorders (Zetzsche et al., 2007), Visser et al. (2014) suggest that previous findings may be explained by comorbid symptoms, rather than aggression per se. Since this is the first study investigating this association in young children, differences in findings may be related to the young age of our sample. The relation between hippocampal volume and aggression in typically developing populations therefore remains an issue that requires further investigation.

Several studies on clinical and non-clinical aggression have reported reduced right ACC and OFC volume or cortical thickness in association with aggression (Ameis et al., 2014; Boes et al., 2008; Ducharme et al., 2011; Yang & Raine, 2009). Our exploratory analyses did not replicate these findings. The present study was the first structural imaging study on aggressive behavior focusing on non-clinical young children. As the (pre)frontal cortex is a brain region that matures later compared to other more primary regions of the brain (e.g., sensorimotor cortex) (Gogtay et al., 2004), the association between aggression and ACC and OFC thickness or surface area may become ‘unmasked’ later in life. Moreover, we used surface-based analyses to study the association between cortical morphometry and aggressive behavior. As many earlier studies have used volume-based morphometric analyses, discrepancies between the present studies and previous literature may stem from the approach used to analyze the data.

Some limitations should be noted. The present study used cross-sectional imaging data and does not provide information on development over time. Furthermore, the MRI procedure (mean age = 7.9 years) was performed at a later time point than the aggressive behavior assessment (mean age = 6.0 years). However, since it has been shown that aggression becomes relatively stable at age 4 (Verhulst & Van der Ende, 1995), we believe that this difference in timing of the assessment should not substantially affect the results. Moreover, analyses were corrected for age at MRI procedure and age at aggressive behavior assessment. Finally, when using a data driven approach there is always the risk of reverse inference. However, even when using such an approach, our results do mesh well with the existing literature. Through bootstrapping we have tried to lower the risk of reporting chance findings.

In conclusion, we are the first to assess the association between brain anatomy and normal variation in childhood aggressive behavior in a large population-based sample using a multiple-informant approach. While several studies have shown associations between aggression and amygdala volume in clinical samples or in adults, we provide novel evidence that aggression is related to decreased amygdala volume also in young typically developing children. We show that childhood aggressive behavior is associated with decreased sensorimotor cortical thickness and widespread decreased right hemisphere gyrification. Moreover, aggressive behavior was associated with cortical thickness in regions that are part of the DMN, with positive associations in girls and negative associations in boys. While the associations with a priori hypothesized regions were small, larger effects were found that were widespread and suggested a more global association with brain morphology. Longitudinal studies are necessary to shed light on the developmental trajectories underlying aggression.

## Electronic supplementary material

Below is the link to the electronic supplementary material.

ESM 1 (DOCX 4.33 mb)
