# Supplementary material for: Brain morphology of childhood aggressive behavior: A multi-informant study in school-age children
Source: Cogn Affect Behav Neurosci. 2015 Mar 24;15(3):564–77. doi: 10.3758/s13415-015-0344-9 (PMC4526589; doi:10.3758/s13415-015-0344-9)
Supplement: Supplementary file 1 — (DOCX 4.33 mb) [file 13415_2015_344_MOESM1_ESM.docx]

Supplementary Table 1

*Aggressive behavior (parental-report and child-report),amygdala and hippocampus volume (n=500)*

|  | |  |  | Combined | |  | Left | |  | Right |  |  |
| --- | --- | --- | --- | --- | --- | --- | --- | --- | --- | --- | --- | --- |
|  | |  |  | B (95% CI) | β | *part r* | B (95% CI) | β | *part r* | B (95% CI) | β | *part r* |
| Amygdala | Parent-report | Baseline adjusted^1^ | | -26.39 (-57.29; 4.51) | -.08 | -.07 | -9.80 (-26.00; 6.41) | -.05 | -.05 | -15.59 (-34.37; 3.19) | -.08 | -.07 |
|  |  | Adjusted^2^ | | -48.71 (-88.99; -8.42) | -.14* | -.10 | -17.37 (-38.41; 3.67) | -.10 | -.07 | -31.15 (-55.62;-6.69) | -.15* | -.11 |
|  | Child-report | Baseline adjusted^1^ | | -13.17 (-49.44; 23.10) | -.03 | -.03 | -3.87 (-22.88; 15.85) | -.02 | -.02 | -8.69 (-30.73; 13.35) | -.04 | -.04 |
|  |  | Adjusted^2^ | | -16.22 (-52.99; 20.54) | -.04 | -.04 | -6.30 (-25.49; 12.88) | -.03 | -.03 | -9.45 (-31.85; 12.95) | -.04 | -.04 |
| Hippo-campus | Parent-report | Baseline adjusted^1^ | | 18.77 (-28.27; 65.81) | .03 | .03 | 14.12 (-11.66; 39.90) | .05 | .05 | 4.15 (-21.98; 30.27) | -.00 | .01 |
|  |  | Adjusted^2^ | | 5.13 (-56.38; 66.65) | .01 | .01 | 4.54 (-28.98; 38.05) | .02 | .01 | 0.50 (-33.71; 34.70) | .01 | .00 |
|  | Child-report | Baseline adjusted^1^ | | -3.99 (-59.14; 51.15) | -.01 | -.01 | 2.99 (-27.34; 33.21) | .01 | .01 | -7.29 (-37.90; 23.32) | -.02 | -.02 |
|  |  | Adjusted^2^ | | -15.57 (-71.38; 40.23) | -.03 | -.03 | -4.97 (-35.40; 25.46) | -.02 | -.02 | -10.42 (-41.53; 20.69) | -.03 | -.03 |

Note. * p < .05 ** p < .01*** p <.001. Amygdala and hippocampus volume were corrected by TBV.

^1^Adjusted for gender and age

^2^Adjusted for gender, age, IQ, gestational age, ethnicity, handedness, attention problems, internalizing problems, prosocial behavior, image quality, maternal education, parental psychopathology, maternal smoking during pregnancy

Supplementary Text 1. Associations between cortical thickness, cortical surface area, and gyrification and parent- and child-reported aggressive behavior

Parent-report aggressive behavior was negatively associated with cortical thickness in a cluster including the right supramarginal and postcentral cortex (1209 mm², max vertex X = 42.9, Y = -27.6, Z = 37.6, p = .004, see Supplementary Figure 2a). More child-report aggressive behavior was related to a thinner cortex in a cluster including the right precentral and caudal middle frontal cortex (1902 mm², max vertex X = 40.2, Y = -10.9, Z = 42.6, p < .001, see Supplementary Figure 2b). For both the parent and the child-report data, none of the clusters in which we found an aggression-by-gender interaction effect survived correction for multiple comparisons.

Parent-reported as well as child-reported aggressive behavior was not associated with cortical surface area. In the left hemisphere, parent-reported aggression was associated with reduced gyrification in a cluster including the pre- and postcentral gyrus, extending to the supramargincal, temporal, and insular cortex (9460 mm², max vertex X = -57.2, Y = -0.1, Z = -9.5, p < .001), as well as a cluster including the precuneus, cuneus, lingal and fusiform cortex, and isthmus of the cingulate cortex (7877 mm², max vertex X = -21.4, Y = -61.3, Z = 8.9, p < .001 .), and a cluster including the rostral middle frontal cortex (2440 mm², max vertex X = -38.1, Y = 50.0, Z = -3.4, p = .01). In the right hemisphere, parent-reported aggressive behavior was associated with reduced gyrification in a cluster including the precental, postcentral, superior parietal, supramarignal, superior frontal and middle frontal cortex (6723 mm², max vertex X = 27.7, Y = -14.4, Z = 60.2, p < .001), as well as in a cluster including the insula, pre- and postcentral cortex, supramarginal and superior temporal cortex (5841 mm², max vertex X = 37.1, Y = -13.0, Z = 1.8, p < .001), and a cluster including the later occipital and lingual cortex, and precuneus (5262 mm², max vertex X = 21.3, Y = -98.7, Z = 5.3, p < .001). The fourth right hemisphere cluster included the bankssts and inferior parietal cortex (2252 mm², max vertex X = 21.3, Y = -98.7, Z = 5.3, p = .02), while a fifth cluster included the right rostral middle frontal and lateral orbitofrontal cortex (2060 mm², max vertex X = 27.7, Y = 57.8, Z = -9.5, p = .04). Gender moderated the association between parent-reported aggression and gyrification in a cluster including the right middle frontal, superior frontal, precentral, and postcentral cortex (5762 mm², max vertex X = 32.6, Y = 18.7, Z = 47.2, p = .009).

Child-reported aggressive behavior was associated with reduced gyrification in a cluster including the left supramarginal, extending to the post- and precentral cortex, and caudal middle frontal and superior frontal cortex (6918 mm², max vertex X = -49.6, Y = -48.0, Z = 44.6, p < .001). In the right hemisphere, child-reported aggression was associated with reduced gyrification in a cluster including the parietal, postcentral and supramarginal cortex (3281 mm², max vertex X = 30.6, Y = -47.5, Z = 44.4, p = .001), as well as in a cluster including the lateral occipital and parietal cortex (2830 mm², max vertex X = 21.3, Y = -98.7, Z = 5.3, p = .006). Gender moderated the association between child-reported aggression and gyrifcation in a cluster including the right postcentral and precentral gyrus (3196 mm², max vertex X = 27.7, Y = -14.4, Z = 60.2, p = .001).


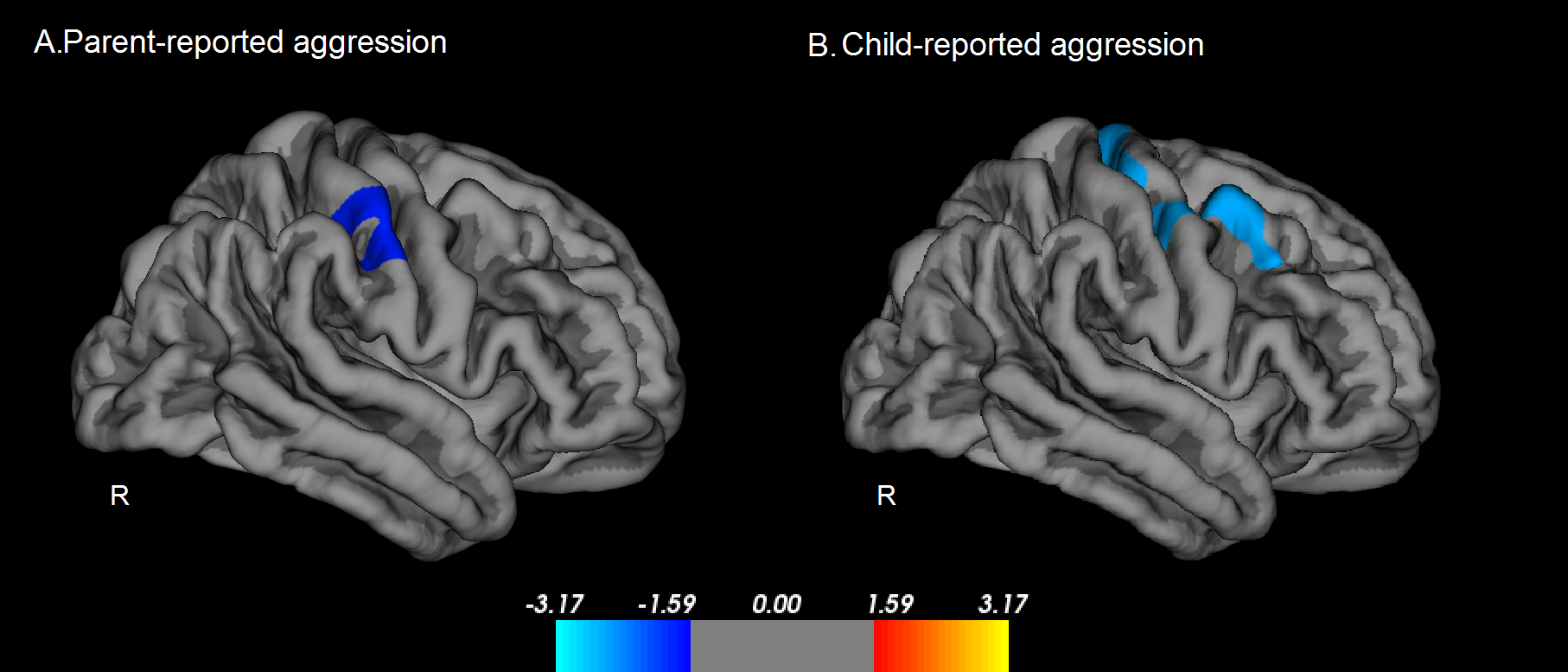


Supplementary Figure 1.

Association between cortical thickness and parent-reported and child-reported aggressive behavior. Sex, age and IQ were used as covariates (Monte Carlo corrected cluster-wise p < .05). Colors represent –log10 *p-value*.


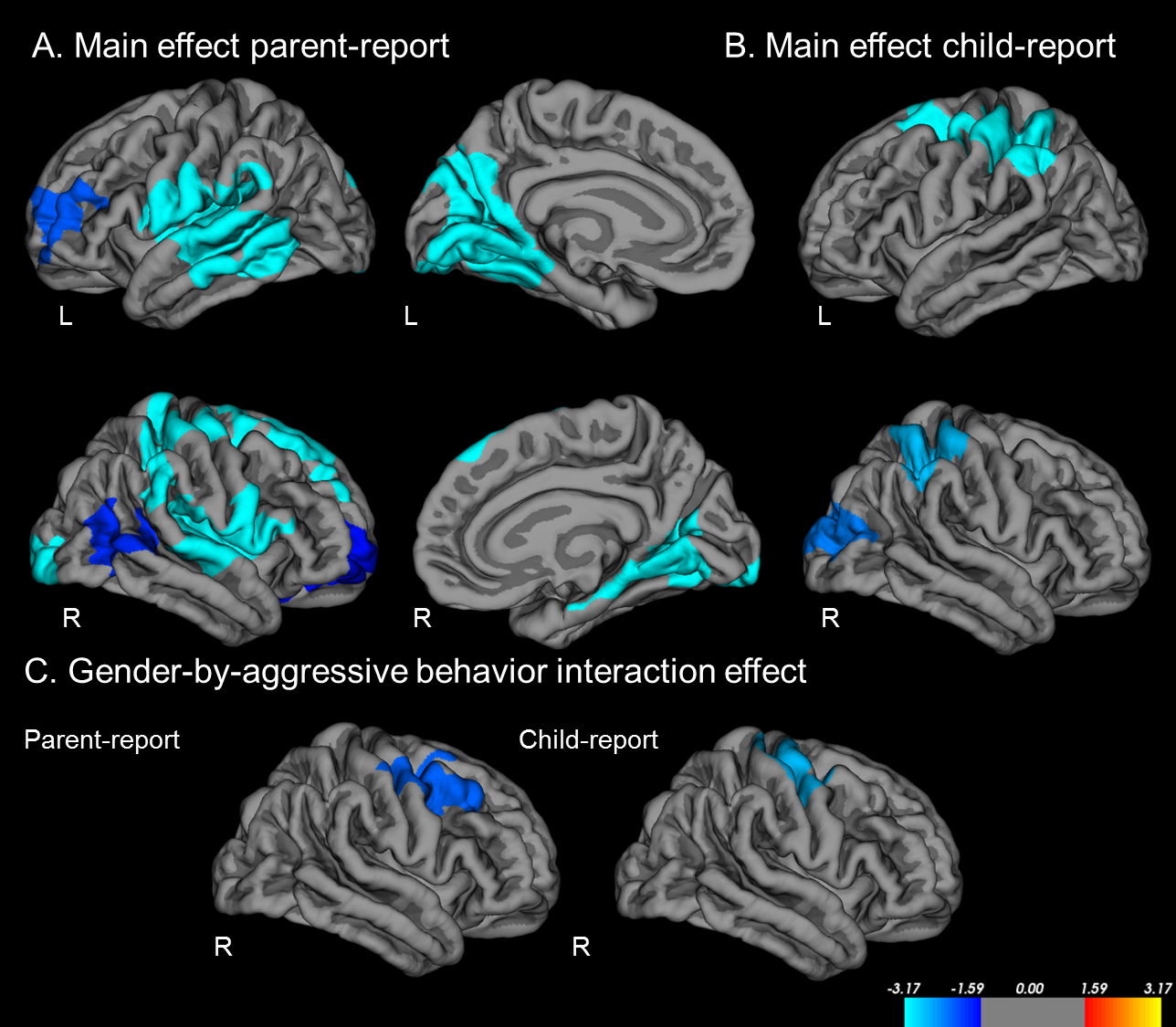


Supplementary Figure 2.

Association between cortical gyrification and parent-reported and child-reported aggressive behavior. Sex, age and IQ were used as covariates (Monte Carlo corrected cluster-wise p < .05). Colors represent –log10 *p-value*.


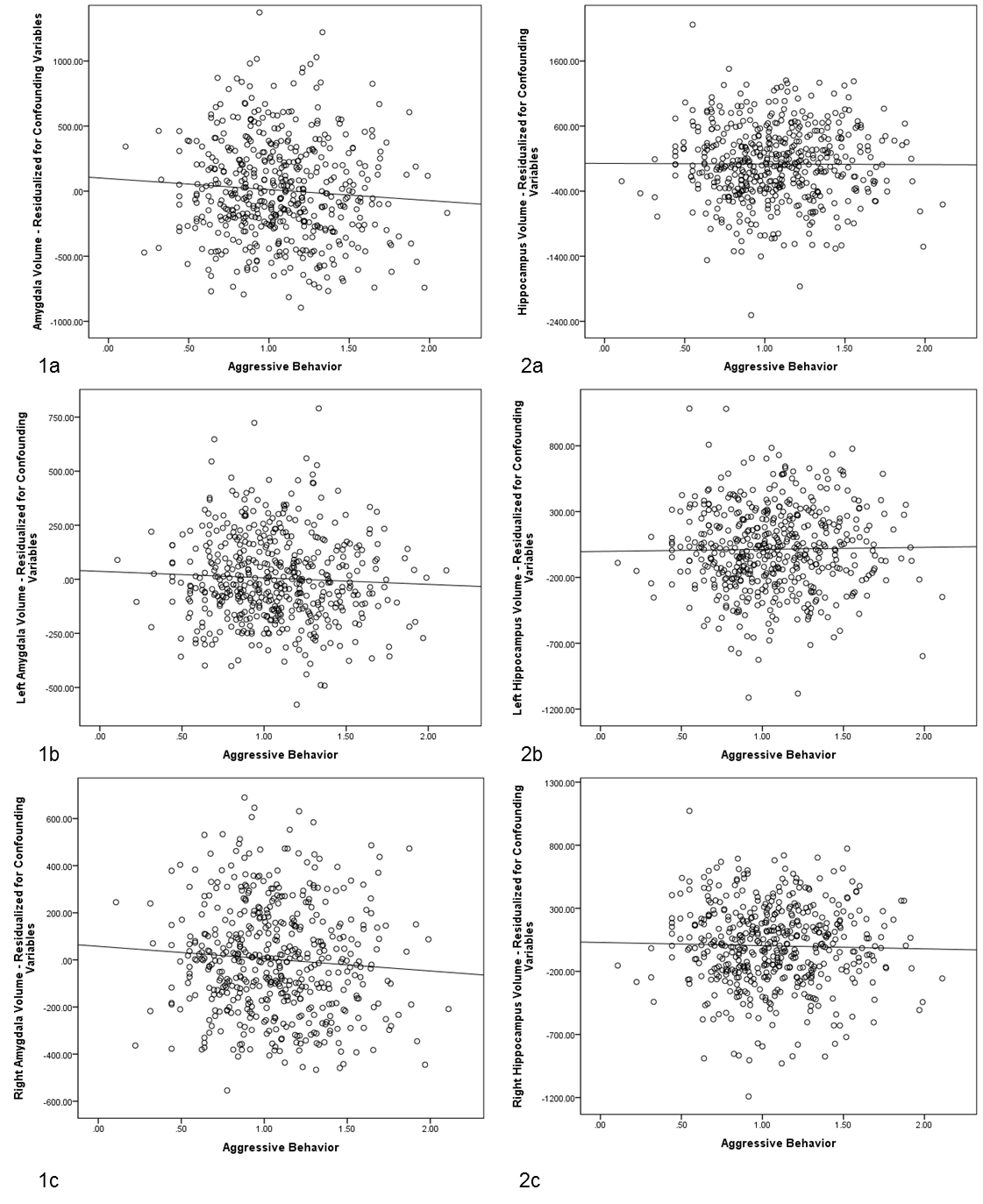


Supplementary Figure 3

Scatterplot of the association between aggressive behavior and amygdala 1a. amygdala volume; 1b. left amygdala volume; 1c. right amygdala volume; 2a. hippocampal volume; 2b. left hippocampus volume; 2c. right hippocampus volume. Amygdala and hippocampal volumes were residualized for total brain volume and confounding variables.


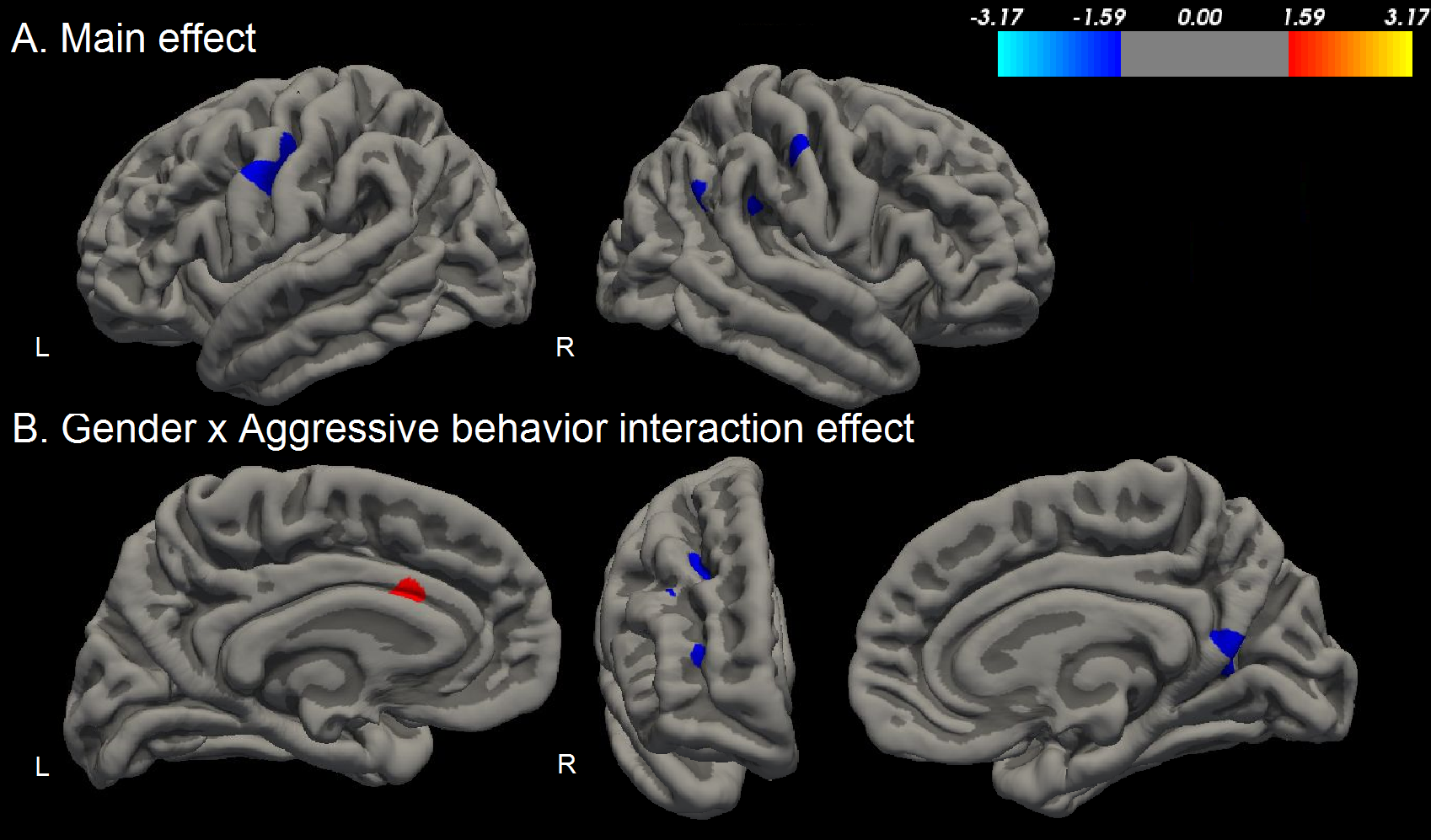
Supplementary Figure 4.

Averaged results of the bootstrapped association between cortical thickness and aggressive behavior. A. Aggressive behavior was related to a thinner cortex in a cluster including the left precentral cortex and a cluster including the right inferior parietal, supramarginal, and postcentral cortex. B. Gender moderates the associations between aggressive behavior and cortical thickness in a cluster including the left ACC, in a cluster including the right middle and superior frontal cortex and in a cluster including the right precuneus. Colors represent –log10 *p-value*.


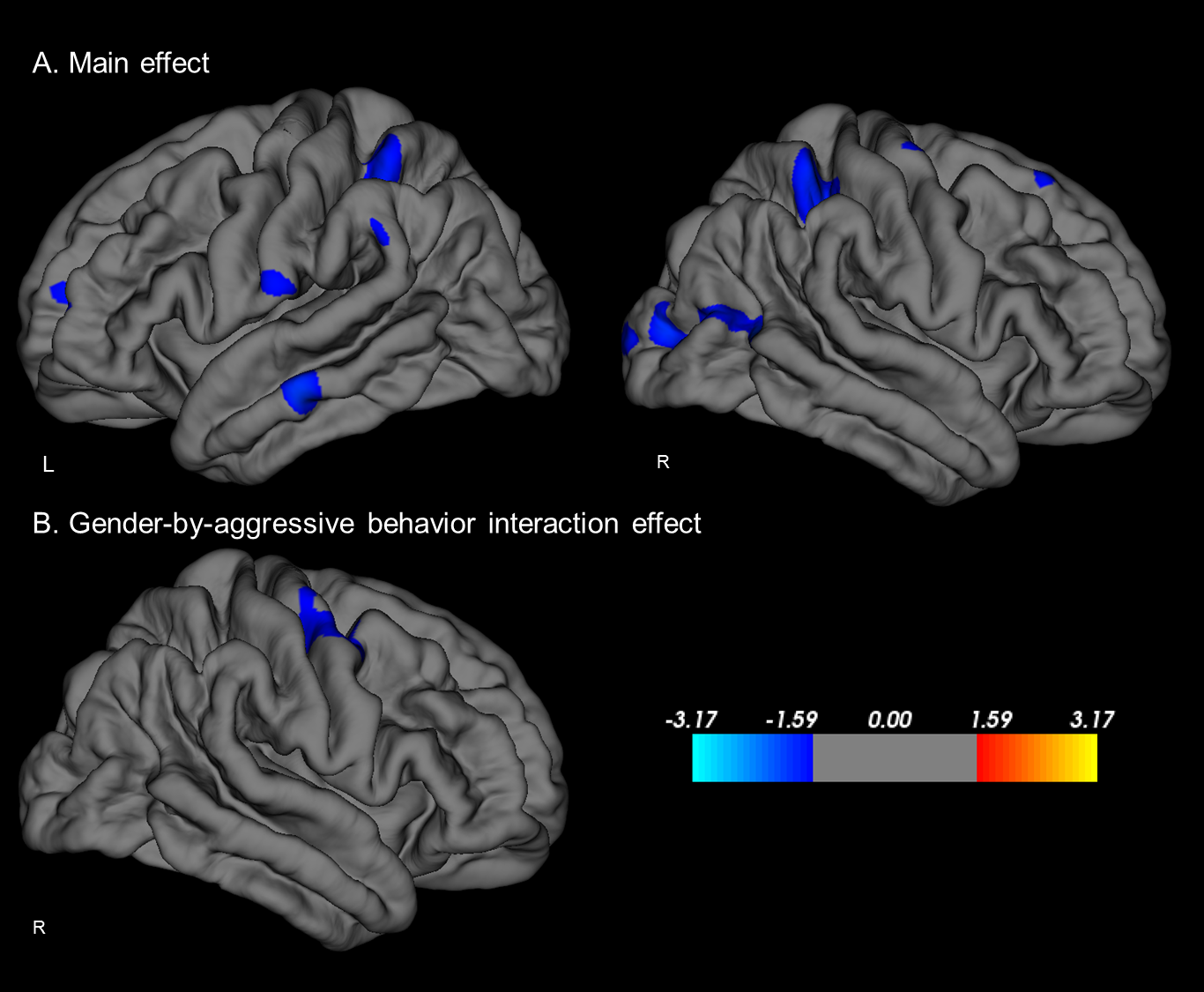
Supplementary Figure 5.

Averaged results of the bootstrapped association between cortical gyrification and aggressive behavior. A. Aggressive behavior was related to a decreased gyrification in the left middle temporal, parietal, postcentral and middle frontal cortex as well as the in the right parietal, lateral occipital, precentral and middle frontal cortex. B. Gender moderates the associations between aggressive behavior and cortical gyrification in a cluster including the right precentral gyrus. Colors represent –log10 *p-value*.
